# Effect of Lipids on Diabetic Retinopathy in a Large Cohort of Diabetic Patients after 10 Years of Follow-Up

**DOI:** 10.3390/jcm12206674

**Published:** 2023-10-22

**Authors:** Pedro Romero-Aroca, Raquel Verges, Jordi Pascual-Fontanilles, Aida Valls, Josep Franch, Joan Barrot, Xavier Mundet, Alex La Torre, Antonio Moreno, Ramon Sagarra, Josep Basora, Eugeni Garcia-Curto, Marc Baget-Bernaldiz

**Affiliations:** 1 Ophthalmology Service, University Hospital Sant Joan, Institut de Investigacio Sanitaria Pere Virgili (IISPV), 43204 Reus, Spain; raquel.verges@salutsantjoan.cat (R.V.); alex.latprre@salutsantjoan.cat (A.L.T.); rsagarraalamo@gmail.com (R.S.); eugenigorg@gmail.com (E.G.-C.); mbaget@gmail.com (M.B.-B.); 2 ITAKA Research Group, Department of Computer Science and Mathematics, Universitat Rovira i Virgili, 43007 Tarragona, Spain; jordi.pascual@urv.cat (J.P.-F.); antonio.moreno@urv.cat (A.M.); 3 Diabetes from Primary Care (DAP)-Cat Group, Unitat de Suport a la Recerca Barcelona, Fundació Institut Universitari per a la Recerca a l’Atenció Primària de Salut Jordi Gol i Gurina (IDIAPJGOL), 08007 Barcelona, Spain; josep.franch@gmail.com (J.F.); joanbarrot@hotmail.com (J.B.); mundetx@gmail.com (X.M.); jbasora.tarte.ics@gencat.cat (J.B.)

**Keywords:** diabetic retinopathy 2, lipids, TC/HDL-cholesterol ratio, No-HDL-C/HDL-cholesterol ratio, fibrates, statins

## Abstract

(1) Background: Diabetic retinopathy (DR) remains the leading cause of low vision and blindness in young adults of working age. Although the most important risk factors—such as the duration of diabetes mellitus (DM) and glycemic control measured by HbA1c—are known, the effects of lipids are not as clear. The aim of the present study is to analyze the effects of lipids on the development of DR. (2) Methods: This is a retrospective study of a population of 175,645 DM2 patients, during the period 2010 to 2020, in which the effects of different lipid factors are studied. (3) Results: The variables that most influenced the development of DR in our study, based on significance and cumulative hazard (CH), were arterial hypertension (CH 1.217, *p* < 0.001), HbA1c levels (CH 1.162, *p* = 0.001), microalbuminuria (CH 1.012, *p* < 0.001), LDL-C cholesterol (CH 1.007, *p* = 0.012), TC/HDL-C index (CH 1.092, *p* < 0.001), No-HDL-C/HDL-C index (CH 1.065, *p* = 0.002), the use of statins (CH 1.001, *p* = 0.005), and body mass index (CH 1.007, *p* < 0.001). (4) Conclusions: LDL-cholesterol, TC/HDL-C, and No-HDL-C/HDL-C indices are related to the development of DR, and there is a protective effect of HDL-cholesterol and the use of fibrates.

## 1. Introduction

Type 2 diabetes mellitus (DM2) affects 10% of the world’s population, and this is expected to increase significantly in the coming decades [1]. With the currently available resources, diabetic retinopathy (DR) cannot be detected in its early stages [2].

In Catalonia, 630,000 people (7.9% of the population) have known DM; however, according to the Di@bet.es study, the total prevalence of DM in Spain is 13.8% (7.9% with known DM, and 7.9% with unknown DM) [3].

The risk factors currently identified for DR are current age, the duration of DM since diagnosis, the treatment undergone for DM (especially in patients treated with insulin), the control of glycemia as measured by the HbA1c levels, associated arterial hypertension, the state of renal function as measured by studying glomerular filtration using the eGFR formula, and the albumin-to-creatinine ratio (ACR) [4,5,6,7]. We must also take into account that patients with more than 5 years of diabetes may have other complications such as nephropathy or cardiovascular pathologies, which can be associated with diabetic retinopathy—this being an indicator of kidney or cardiovascular disease.

Although cardiovascular diseases are associated with lipid alterations, for diabetic retinopathy development, the importance of lipid levels is controversial.

Although pathophysiologically there may be a cause–effect relationship at the local level of the retina, the accumulation of lipid aldehydes and advanced lipoxidation end products (ALEs) gives rise to the modification of the structure of cellular proteins, leading to alterations in the barrier and retinal hematoma, and also allows the activation of neutrophils and macrophages that have been observed in the retina—especially at the macular level. Likewise, secondary reactions to lipid peroxidation have been associated with diabetic cardiovascular pathology [7].

In contrast, the relationship between blood lipid levels and DR is not clear [8]. Some studies have investigated the association between the presence of hard exudates and blood cholesterol levels [9,10], and others have reported on treatment with statins or fibrates [11,12,13,14,15].

Through a retrospective study involving 175,645 patients over 11 years, the present study aims to determine the influence of the levels of various lipids, lipid indices, and the treatment of dyslipidemia with statins or fibrates on the development of diabetic retinopathy.

## 2. Materials and Methods

### 2.1. Setting

The population of Catalonia is 7.566 million. A total of 630,000 (7.9%) patients have been diagnosed with DM2 (56.9% men, 43.1% women). Prevalence increases with age—especially in patients over 55 years [3].

### 2.2. Sample Size

We included retrospective data from the Electronic Health Records (EHR) of 175,645 DM2 patients, provided by SIDIAP. The primary care SIDIAP database (“Sistema d’informació pel Desenvolupament de la Recerca a Atenció Primària”) was used as the data source. The SIDIAP database routinely collects pseudo-anonymized healthcare data from users who attend the primary healthcare centers of the Catalonian Health Institute (Institut Català de la Salut-ICS). The ICS is the largest healthcare provider in Catalonia (Barcelona, Spain) and covers about 80% (5,564,292 persons) of the Catalonian population. The SIDIAP database contains various patient data, such as visits with healthcare professionals, diagnostic codes, demographic information, clinical variables, laboratory test results, prescriptions, referrals to specialists and hospitals, and medication dispensed in pharmacies. For this analysis, data from an 11-year period were extracted (2010–2020, inclusive).

### 2.3. Inclusion Criteria

The following are the inclusion criteria of this study: Patients with type 2 DM.

### 2.4. Exclusion Criteria

The following are the exclusion criteria of this study:

- Patients with type 1 DM.
- Patients included in diabetes group III and other specific types (i.e., diseases of the exocrine pancreas, endocrinopathy, genetic defects of β-cell function, genetic defects in insulin action).
- Patients included in diabetes group IV and gestational diabetes mellitus (GDM).
- Patients who did not have a complete EHR.
- Patients with diabetic retinopathy (DR) at inclusion.

### 2.5. Epidemiological Risk Factors Included in the Study

The following are the epidemiological risk factors included in this study:

- Age and sex.
- Duration of DM since diagnosis.
- DM treatment (at three levels: diet, oral hypoglycemics, and insulin).
- Arterial hypertension, which is indicated by a systolic/diastolic (normal value = 140/90 mm Hg) measurement according to the report of the sixth joint National Committee on the Prevention, Detection, Evaluation, and Treatment of High Blood Pressure; and when the patient is taking anti-hypertension medications.
- Levels of glycosylated hemoglobin (HbA1c), defined according to the American Diabetes Association recommendation.
- BMI levels measured in kg/m^2^.
- Study of renal status, determining ACR (albumin-to-creatinine ratio; values in mg/g in 24 h urine). Values, normal if <30 mg/g, microalbuminuria if 30 to 299 mg/g and macroalbuminuria if >300 mg/g. In addition, determining the estimated glomerular filtration rate (eGFR), as measured by CKD-EPI (values in mL/min).
- Levels of Total-cholesterol (TC), LDL-C-cholesterol (LDL-C), VLDL-C-C-cholesterol (VLDL-C-C), HDL-C-cholesterol (HDL-C), and triglycerides (TG).
- Lipid indices, Castelli index I Total cholesterol/HDL-cholesterol (TC/HDL-C), Castelli index II LDL-cholesterol/HDL-cholesterol (LDL-C/HDL-C), No-HDL cholesterol/HDL-cholesterol (No-HDL-C/HDL-C) index, and triglycerides/HDL-cholesterol (TG/HDL-C) index.
- Use of statins or fibrates.

### 2.6. Description of the Clinical Data Collection

All patients underwent the same type of test at their nearest laboratory, using the same criteria in all cases. For each patient, it was required that, at least every year, they underwent a complete analysis that included the variables analyzed in the present study.

### 2.7. Diagnosis of Diabetic Retinopathy

Data on DR were extracted from the EHR; the presence or absence of DR and the type of DR were noted. The diagnosis of DR was based on retinographies performed on patients with DM2. In most centers, the camera used for DR screening is the Topcon NW400 retinal cameras. DR was classified according to standard international DR classifications [16].

### 2.8. Ethical Adherence

The study was carried out in accordance with local legal requirements (approval number Ref. CEIM: 028/2018, by the local ethical committee of Hospital Universitari Sant Joan de Reus) and with the revised guidelines of the Declaration of Helsinki. The nature of the study was explained to all patients, who provided written consent for their participation. The study was approved and supported by Instituto de Salud Carlos III (IISCIII), Spain, number PI21/00064.

### 2.9. Statistical Methods

The data collected in the study started from 2010 and ended in 2020. Each year, there was at least one analysis of all the study data on each patient included in the study. At the time of inclusion, no patient had diabetic retinopathy. The statistical study was carried out from the time of inclusion of the patient (year 2010) until the patient developed diabetic retinopathy. In the event that the patient did not do so, the patient data included in the study were all measurements collected from the year 2010 to the year 2020.

Study power: We assumed a significance level of 0.05; for the desired power of 90%, as known from previous study estimates of the sample size needed to detect patients with any type of diabetic retinopathy—the prevalence of which is 15%—a minimum of 97,452 patients with diabetes mellitus were needed.

Data were analyzed using SPSS version 22.0 (IBM^®^ Statistics, Chicago, IL, USA). We included the following independent risk factors: age (as current age); sex; DM duration; DM treatment; HbA1c; arterial hypertension; body mass index; microalbuminuria; glomerular filtration rate, as measured via CKD-EPI; the values of total cholesterol, LDL-C, HDL-C, and VLDL-C-C, and triglycerides; statin treatment; fibrates treatment; the Castelli I and II risk indices; no-HDL-C/HDL-C; and TG/HDL-C. All parameters were assessed according to established procedures. A *p*-value of less than 0.05 was considered to indicate statistical significance.

A descriptive statistical analysis of quantitative data was performed by the determination of the mean and standard deviation. For qualitative data, we analyzed the frequency and percentage in each category.

The univariate study was carried out using the two-sample Student’s *t*-test to compare quantitative variables, and we used the Chi-squared table for qualitative data.

We carried out a multivariate study, calculating the COX survival statistic by determining the mean, the survival value ± standard deviation, the cumulative hazard with 95% CI, and the degree of statistical significance. The results were interpreted based on their statistical significance and cumulative hazard (CH).

In the COX survival study, the determination of the cumulative hazard is basic, as it can have three values for the same degree of statistical significance. A cumulative hazard value of 1.000 means that it is a neutral value that does not influence the independent variable (in our case, the formation of DR). If the cumulative hazard value is greater than 1.000, it is significant, and therefore the risk factor positively affects the occurrence of DR. If the cumulative hazard value is less than 1.000, the risk factor induces a negative or protective effect against the appearance of DR. It is important to take into account the 95% confidence interval that we have calculated for all the variables.

Three examples of how the values can be interpreted:

- The CH value is CH1.002 (≥1.000) and the interval is 1.000–1.023; then, the effect is positive/protective.
- The CH value is CH 0.976 (<1.000) and the interval is 0.954–0.999; then, the effect is negative.
- The CH value is CH1.002 (≥1.000) and the interval is 0.954–1.023; then, the effect is neutral.

## 3. Results

The sample size was 175,645 patients, of which 99,991 were men (56.9%) and 75,654 were women (43.1%). According to race, 4910 patients (2.75%) were non-Caucasian, of which 2763 were African (1.57%) and 2147 were Asian (1.18%). The mean age was 67 ± 11.13 years (range: 30–104). The mean duration of DM since diagnosis was 7.67 ± 6.04 years (range: 0–57). Regarding treatment, 24,897 were treated with diet (14.2%), 129,767 with oral hypoglycemic agents (73.9%), and 20,981 with insulin (11.9%). A total of 57,096 patients had arterial hypertension (32.5%), 52,797 were treated with statins (30.1%), and 6567 were treated with fibrates (3.7%). After 11 years of follow-up, a total of 26,156 patients developed DR (14.9%). Regarding DR type, 17,534 patients had mild DR (10%), 6160 had moderate DR (3.5%), 1084 had severe DR (0.6%), 717 had proliferative DR (0.4%), and 661 had diabetic macular edema (0.4%).

### Univariate Study

Table 1 shows the differences between patients with or without diabetic retinopathy at the end of the study in terms of risk factors and their statistical significance.

According to the univariate study, only age and sex were not significant. The other variables—treatment and duration of DM, body mass index, presence of arterial hypertension, HbA1c levels, ACR, and glomerular filtration as measured by CKD-EPI—were significant.

Table 2 shows the lipid measurements at the end of study, with significant mean differences in the values of LDL cholesterol (LDL-C) and HDL cholesterol (HDL-C). The differences in Total-cholesterol (TC), VLDL cholesterol (VLDL-C), and triglycerides (TG) were not significant.

Regarding the study of lipid indices, the Castelli I index (TC/HDL-C) and the No-HDL-C/HDL-C index were significant at *p* < 0.001, and the differences in the lipid index, Castelli II (LDL-C/HDL-C), and the TG/HDL-C index were not significant.

Regarding treatment with statins and fibrates, both were significant when applying the Chi-squared test. For statins, there was a positive risk of DR at an odds ratio of 1.16, with a significance of *p* < 0.001. For fibrates, there was a negative risk of DR at an odds ratio of 1.148, with a significance of *p* < 0.001.

Table 3 shows the survival function of COX for the different variables. Although many of the variables studied were significant in the COX regression, we must look to the cumulative hazard (CH) to know the power of each variable in the risk of DR. If the CH is above 1.000, it implies a positive effect for the development of DR; if it is below 1.000, it implies a protective effect—that is, DR is not induced. If the value is 1.000, the effect on the independent variable is neutral. At *p* < 0.001, a CH value of 1.000 is not significant.

The variables that most influenced the development of DR in our study—based on the CH values—were the presence of arterial hypertension (CH 1.217, *p* < 0.001), elevated levels of HbA1c (CH 1.162, *p* = 0.001), microalbuminuria (CH 1.012 *p* < 0.001), LDL-C cholesterol (CH 1.007, *p* = 0.02), Castelli index I (TC/HDL-C, CH 1.092, *p* < 0.001), No-HDL-C/HDL-C ratio (CH 1.065, *p*= 0.002), the use of statins (CH 1.001, *p* < 0.005), and high body mass index (CH 1.007, *p* < 0.001).

Significant variables with CH values lower than 1.000—and therefore demonstrating a negative effect on the development of DR—were current age (CH 0.971, *p* < 0.001), sex (CH 0.960, *p* = 0.004), glomerular filtration rate measured by eGFR (CH 0.996, *p* < 0.001), HDL-C cholesterol (CH 0.996, *p* = 0.001), and the use of fibrates (CH 0.907, *p* = 0.003). VLDL-C-C cholesterol (CH 0.994, *p* = 0.905), total cholesterol (CH 1.001, *p* = 0.136), triglyceride levels (CH 1.000, *p* = 0.632), and Castelli index II (CH 0.880, *p* = 0.116) were not significant.

For the TG/HDL-C index, with a CH value of 0.006 and *p* = 0.660—although the CH value was 0.996 and therefore could indicate a negative effect—the confidence interval of the CH was between 0.994 and 1.004, which includes the value 1.000 in the CI and which is considered to have a neutral effect on the independent variable—that is, the development of DR.

## 4. Discussion

The main strength of the present study is the sample size. A total of 175,645 patients with type 2 DM is representative of the geographical area of study; additionally, the study was carried out over a period of 11 years—from 2012 to 2022, the period for which data are available. Our results agree with other studies with respect to the already-known risk factors; current age, sex, duration of DM since diagnosis, and treatment of DM were all significant, as well as high levels of HbA1c, a high body mass index, and the presence of arterial hypertension. Similarly, renal function was studied through the ACR and eGFR parameters.

Lipid factors in routine clinical practice. There were significant differences in the LDL-C cholesterol lipid fraction—which has a significant effect on the development of DR (CH 1.007, *p* = 0.001)—and HDL-C cholesterol, which has a protective effect from the development of DR (CH 0.996, *p* = 0.001). In the case of LDL-C, a CH value greater than 1.000 therefore has a positive effect on the development of DR, and in the case of HDL-C, a value of less than 1.000 has a negative effect on the formation of DR. The values for TC, VLDL-C-C, and triglycerides were not significant in the present study. If we compare our results with those of other studies, the relationship between lipids and the development of DR is not proven. Early studies that were carried out reported a positive relationship between the number of hard exudates present in the retina and lipid levels. The WESDR study (Wisconsin Epidemiological Study of Diabetic Retinopathy), in its 13th publication (1991) [10], found a positive relationship with total cholesterol levels. Another publication by the ETDRS (Early Treatment Diabetic Retinopathy Study) group, in their 22nd report (1996) [11], concluded that patients with high levels of total cholesterol and LDL-C cholesterol were at higher risk of being diagnosed with DR in the presence of hard exudates—as in the previous study. More recently, other studies such as the MESA (The Multi-Ethnic Study of Atherosclerosis) were not able to determine any association between the presence of DR and the levels of HDL-C-cholesterol, LDL-C-cholesterol, or triglycerides [17]. In a recent review of articles published on hyperlipidemia and its relationship with DR, the authors [9] established that such a relationship is discordant; thus, more studies are needed for clarity.

The use of lipid indices as atherogenic indices in cardiovascular diseases. Our results indicate that the Castelli I risk index—which is determined by the TC/HDL-C ratio—is significant in the development of DR, with a CH value of 1.092 and *p* < 0.001. Likewise, the result for the non-HDL-C/HDL-C index was positive, with a CH value of 1.065 and *p* = 0.002. TG/HDL-C indices and the Castelli II risk index are determined by the LDL-C/HDL-C fraction, but were not significant (*p* = 0.116).

The use of statins or fibrates to treat dyslipidemias. The study of cardiovascular risk indices allows us to explore a new field that might help us to better detect the risk of developing DR. Evaluating lipid indices is a relatively recent development in the study of patients with cardiovascular risk, since LDL-C values have exclusively turned out to not be optimal for assessing risk—especially in patients at intermediate risk [18]. The Framingham Heart Study and the Coronary Primary Prevention Trial [19] demonstrated that the TC/HDL-C and LDL-C/HDL-C indices are good predictors of cardiovascular disease—especially in cases of hypertriglyceridemia, in which it is preferable to use the values of TC/HDL-C.

Of the four ratios that we evaluated, only two yielded positive results. One of these was the TC/HDL-C index or Castelli I risk index, which is a mathematical formula that calculates the risk of a person suffering atherosclerosis based on their cholesterol levels. We were able therefore to determine, or predict, the probability that a person’s arteries will clog up if they go untreated. 

As reference values, there is a risk of atherosclerosis at 3.5 in men and <3 in women. In the present study, we found that patients of both sexes had levels higher than 3.5 (Table 2), which indicates a positive result in its relationship with the development of DR. Regarding the Castelli II index, which is the relationship between LDL-C/HDL-C, the reference values are <2.5 in men and <2 in women. In the present study, the values of this index were below 2.5 in men and above 2 in women, and so this was not significant in the development of DR.

The other index that was found to be significant in our study was the No-HDL-C/HDL-C index. This is calculated by subtracting HDL-C from TC, and represents—in addition to LDL-C—the rest of the cholesterol contained in atherogenic lipoproteins; these include the apolipoprotein B (apoB; formed by LDL-C, VLDL-C, and intermediate density lipoproteins) and lipoprotein(a). This therefore gives us data on the levels of ApoB lipoprotein, which belongs to the group of complex lipoproteins. There are many classes of apolipoproteins, of which Apo-A1 is the major anti-atherogenic HDL protein component, which has anti-inflammatory and antioxidant effects. Apo-B is the main apolipoprotein for intermediate density lipoprotein (LDL-C) and VLDL, and is responsible for the transport of these lipids to peripheral tissues such as the retina, exerting an atherogenic effect in the blood vessels [9,20]. Very little data compare the usefulness of non-HDL-C and these lipoprotein ratios together with conventional lipid measurements when attempting to detect subclinical early-stage atherosclerosis in T2DM.

Regarding the effect of treating dyslipidemia using statins or fibrates, we found that statins have little or no effect on the development of DR, with a CH value of 1.001—significant within the limit at *p* = 0.05. These results agree with the ACCORD study [21], which reported that the use of statins had no effect on the development of DR. More recent studies have found a possible protective effect against the development of DR from the use of statins. The Danish Patient Registry, considering information about drug use from the Danish Registry of Medicinal Product Statistics [22], showed that patients treated with statins before the diagnosis of DM had a lower incidence of DR. Likewise, the Taiwan National Health Insurance Research Database study [23] showed that the use of statins decreased the risk of DR and the need for its treatment. Finally, the Japan Diabetes Complication and its Prevention Prospective (JDCP) study [24] showed that the use of statins and fibrates was associated with a lower risk of developing non-proliferative DR.

Regarding the use of fibrates, the reference study is FIELD (Fenofibrate Intervention and Event Lowering in Diabetes) [13], which determined that the effect of fibrates on the pathogenesis of DR was not related to their effect on triglycerides or other lipoproteins (HDL-C or LDL-C). It was shown to have an independent effect, possibly related to something specific at the level of the retina. From the ACCORD study, a sub-study of patients undergoing treatment with fenofibrate in addition to simvastatin (ACCORD-eye) was developed [25]. Two groups were evaluated: one treated with simvastatin + placebo and the other treated with simvastatin + fenofibrate. The incidence of DR in the group treated with fenofibrate was less than in the placebo group, with the incidence of DR at 4 years being 6.5% vs. 10.2%, respectively. That study also demonstrated that there was a dissociation between the protective effect of fenofibrate, independent of the values of triglycerides, HDL-C cholesterol, and the LDL-C/HDL-C ratio [26].

The limitations of the present study include that it is retrospective and has limited generalizability to other populations. Although we have all the data on the different variables for the patients studied, a prospective study in which we can ensure that we have all the correct data will always be superior. Another weakness is that data were not available on other atherogenic indices, such as apolipoprotein levels. Although there is an association with the occurrence of DR, these measurements are not routine in clinical practice.

## 5. Conclusions

The present study confirms that the effect of lipids on the development of DR remains in doubt due to the low statistical significance of the values determined in routine clinical practice. We were only able to demonstrate that LDL-cholesterol exhibits a positive significance in DR development and that HDL-C has a protective effect on the development of DR.

Measuring lipid indices would appear to be an interesting consideration for future studies, as we have demonstrated that the TC/HDL-C and No-HDL-C/HDL-C indices are related to the development of DR.

We were not able to demonstrate any effect of statins on DR, but there is a protective effect from the use of fibrates that is independent of their effect on triglycerides.

## Figures and Tables

**Table 1:** Demographic data at the end of study and univariate statistical study. * Statistical Student’s *t*-test, ** Chi-squared test F = Fisher–Snedecor distribution. DM duration in years *** = duration of DM since diagnosis. There was significance for DM duration, DM treatment (insulin versus diet and oral agents), arterial hypertension, body mass index, HbA1c levels, albumin to creatinine ratio, and glomerular filtration rate (CKD-EPI).

	With DR	Without DR	F Value/Odds Ratio	Significance
Mean age in years	67.42 ± 11.19	66.92 ± 11.12	F = 1.078	0.299 *
Men	14876 (56.87%)	85115 (55.09%)		
Women	11280 (43.13%)	64374 (44.91%)	OR = 1.003	0.427 **
DM duration in years ***	9.36 ± 6.73	7.37 ± 5.86	F = 410.3	<0.001 *
DM treatment				
Diet	2258 (8.63%)	22639 (14.65%)		
Oral agents	18208 (69.61%)	111559 (72.21%)		
Insulin	5690 (21.75%)	15291 (9.86%)	OR = 3.08	<0.001 **
Arterial hypertension	9860 (37.69%)	47236 (31.59%)	OR = 1.31	<0.001 **
Body mass index	29.85 ± 5.24	30.1 ± 5.32	F = 5.538	0.019 *
HbA1c in %	7.75 ± 1.59	7.17 ± 1.34	F = 1646.34	<0.001 *
ACR (mg/24 h)	45.1 ± 193.19	23.55 ± 119.51	F = 1483.88	<0.001 *
EGFR (mL/min)	73.35 ± 18.05	75.43 ± 16.47	F = 469.86	<0.001 *

**Table 2:** Univariate statistical study of lipids. * Statistical Student’s *t*-test, ** Chi-squared test, F = Fisher–Snedecor distribution.

Lipid Variable	With DR	Without DR	F Value	Significance
Cholesterol total mg/dL	181.92 ± 37.4	184.09 ± 37.08	F = 0.724	0.395 **
Cholesterol LDL-C mg/dL	105.53 ± 31.93	99 ± 31.91	F = 0.558	<0.001 **
Cholesterol HDL-C mg/dL	48.01 ± 11.41	48.43 ± 11.28	F = 7.16	<0.001 **
Cholesterol VLDL-C-C mg/dL	29.33 ± 13.68	30.12 ± 13.63	F = 0.03	0.855 **
Triglycerides mg/dL	153.35 ± 84.35	158.94 ± 230.81	F = 1.33	0.248 *
Use of statins	8588 (32.83%)	44209 (29.57%)	OR = 1.164 *	<0.001 **
Use of fibrates	856 (3.27%)	5711 (3.82%)	OR = 1.148 **	<0.001 **
Castelli index I (Cholesterol total/HDL-C)	Men 3.98 ± 1.06	Men 4.03 ± 1.05	F = 6.54	<0.001 **
	Women 3.78 ± 0.96	Women 3.82 ± 0.94	F = 6.39	
Castelli index II (Cholesterol LDL-C/HDL-C)	Men 2.26 ± 0.78	Men 2.32 ± 0.81	F = 2.61	*p* = 0.094
	Women2.15 ± 0.74	Women 2.19 ± 0.74	F = 2.98	
Ratio NO-HDL-C/HDL-C	8.89 ± 1.02	2.94 ± 1.01	F = 6.545	*p* = 0.001 **
Ratio TG/HDL-C	3.53 ± 2.52	3.61 ± 5.79	F = 0.487	*p* = 0.485 **

**Table 3:** COX regression. SE—standard error.

	Mean	Survival	SE	Cumulative Hazard	IC95%		Significance
Age	67.001	−0.030	0.001	0.971	0.969	0.972	<0.001
Sex	0.431	−0.039	0.014	0.960	0.935	0.986	0.002
DM treatment	0.978	−0.382	0.016	0.682	0.62	0.704	<0.001
Body mass index	30.069	0.007	0.001	1.007	1.004	1.009	<0.001
Arterial hypertension	0.325	0.196	0.013	1.217	1.186	1.248	<0.001
HbA1c	7.265	0.150	0.004	1.162	1.153	1.172	<0.001
ACR	26.765	0.000	0.000	1.012	1.000	1.018	<0.001
CKD-EPI	75.128	−0.004	0.000	0.993	0.991	0.995	<0.001
Cholesterol total	183.473	0.002	0.000	1.001	0.998	1.002	0.136
Cholesterol LDL-C	105.095	0.007	0.001	1.007	1.006	1.009	0.02
Cholesterol HDL-C	48.370	−0.004	0.001	0.996	0.994	0.998	0.001
Cholesterol VLDL-C-C	30.008	−0.005	0.001	0.995	0.994	0.997	0.905
Triglycerides	158.115	0.000	0.000	1.000	0.998	1.002	0.632
Cholesterol Total/HDL-C (Castelli index I)	3.941	0.088	0.019	1.092	1.052	1.134	<0.001
Cholesterol LDL-C/HDL-C (Castelli index II)	2.259	0.101	0.029	0.880	0.750	1.032	0.116
Ratio No-HDL-C/HDL-C	2.941	0.175	0.057	1.191	1.065	1.333	0.002
Ratio TG/HDL-C	3.607	−0.001	0.003	0.999	0.994	1.004	0.660
Use of statins	0.301	0.97	0.013	1.002	1.001	1.003	0.05
Use of fibrates	0.037	−0.104	0.035	0.907	0.846	0.973	0.003

## Data Availability

Not applicable.
